# Recurrence of pseudomyxoma peritonei after cytoreductive surgery and hyperthermic intraperitoneal chemotherapy

**DOI:** 10.1002/bjs5.97

**Published:** 2018-09-27

**Authors:** F. Mercier, F. Dagbert, M. Pocard, D. Goéré, F. Quenet, R. Wernert, F. Dumont, C. Brigand, G. Passot, O. Glehen, the RENAPE Network, J. Abba, K. Abboud, M. Alyami, C. Arvieux, G. Averous, N. Bakrin, G. Balagué, V. Barrau, H. Ben Rejeb, J. M. Bereder, I. Berton Rigaud, F. Bibeau, I. Bonnefoy, D. Bouzard, I. Bricault, C. Caramella, S. Carrère, C. de Chaisemartin, M. Chassang, A. Chevallier, T. Courvoisier, P. Dartigues, A. Dohan, C. Eveno, M. Faruch Bilfeld, G. Ferron, J. Fontaine, L. Fournier, E. Gabiache, J. Gagniere, D. Geffroy, L. Ghouti, F. N. Gilly, L. Gladieff, A. Guibal, J. M. Guilloit, F. Guyon, B. Heyd, C. Hoeffel, C. Hordonneau, S. Isaac, P. Jourdan Enfer, R. Kaci, R. Kianmanesh, C. Labbé Devilliers, J. Lacroix, B. Lelong, A. Leroux Broussier, Y. Lherm, R. Lo Dico, G. Lorimier, C. Malhaire, F. Marchal, P. Mariani, E. Mathiotte, P. Meeus, E. Mery, S. Msika, C. Nadeau, S. Nougaret, P. Ortega Deballon, B. Paquette, O. Pellet, P. Peyrat, D. Pezet, N. Pirro, M. Pocard, F. Poizat, J. Porcheron, P. Rat, P. Rousselot, P. Rousset, H. Senellart, M. Serrano, V. Servois, O. Sgarbura, A. Skanjeti, M. Svrcek, R. Tetreau, E. Thibaudeau, Y. Touchefeu, J. J. Tuech, S. Valmary Degano, D. Vaudoyer, S. Velasco, V. Verriele Beurrier, L. Villeneuve, F. Zinzindohoue

**Affiliations:** ^1^ Department of Digestive Surgery, Centre Hospitalier Lyon Sud, Université Claude Bernard Lyon 1, Lyon, France; ^2^ Equipe Mixte de Recherche 3738, Université Claude Bernard Lyon 1, Lyon, France; ^3^ Surgical Oncological and Digestive Unit, Lariboisière University Hospital, Paris, France; ^4^ Department of Surgery, Gustave Roussy Institute, Villejuif, France; ^5^ Department of Surgical Oncology, Institut du Cancer de Montpellier, Montpellier, France; ^6^ Department of Surgical Oncology, Institut de Cancérologie de l'Ouest (ICO) Paul Papin Cancer Centre, Angers, France; ^7^ Department of Surgical Oncology, ICO René Gauducheau Cancer Centre, St Herblain, France; ^8^ Department of Digestive Surgery, Hautepierre University Hospital, Strasbourg, France; ^9^ Department of Digestive Surgery, Centre Hospitalier Universitaire de Montreal, Montreal, Quebec, Canada; ^10^ Department of Digestive Surgery Grenoble University Hospital Grenoble France; ^11^ Department of General Surgery, Centre Hospitalier Universitaire Jean Monnet University Saint Étienne France; ^12^ Department of Digestive Surgery Lyon Sud University Hospital Lyon France; ^13^ Department of Digestive Surgery Grenoble University Hospital Grenoble France; ^14^ Department of Pathology Hautepierre University Hospital Strasbourg France; ^15^ Department of Digestive Surgery Lyon Sud University Hospital Lyon France; ^16^ Department of Radiology Claudius Regaud Institute Toulouse France; ^17^ Department of Radiology Centre Cardiologique du Nord, Saint Denis France; ^18^ Department of Pathology Bergonie Institute Bordeaux France; ^19^ Department of Digestive Surgery Archet 2 University Hospital Nice France; ^20^ Department of Digestive Oncology, Institut de Cancérologie de l'Ouest (ICO) René Gauducheau Cancer Centre Saint Herblain France; ^21^ Department of Pathology Côte de Nacre University Hospital Caen France; ^22^ Department of Digestive Surgery Lyon Sud University Hospital Lyon France; ^23^ Department of Surgery Louis Mourier University Hospital Colombes France; ^24^ Department of Radiology Grenoble University Hospital Grenoble France; ^25^ Department of Radiology Gustave Roussy Institute Villejuif France; ^26^ Department of Surgical Oncology, Val d'Aurelle Montpellier Cancer Centre Montpellier France; ^27^ Department of Surgical Oncology Paoli Calmettes Institute Marseilles France; ^28^ Department of Radiology Archet 2 University Hospital Nice France; ^29^ Department of Pathology Archet 2 University Hospital Nice France; ^30^ Department of Digestive Surgery Poitiers University Hospital Poitiers France; ^31^ Department of Pathology Gustave Roussy Institute Villejuif France; ^32^ Department of Radiology Lariboisière University Hospital Paris France; ^33^ Surgical Oncological and Digestive Unit Lariboisière University Hospital Paris France; ^34^ Department of Radiology University Hospital of Toulouse Toulouse France; ^35^ Department of Surgical Oncology, IUCT Oncopole Toulouse France; ^36^ Department of Pathology Lyon Sud University Hospital Lyon France; ^37^ Department of Radiology, Georges Pompidou European Hospital Paris France; ^38^ Department of Radiology Claudius Regaud Institute – l'Institut Universitaire du Cancer de Toulouse (IUCT) Toulouse France; ^39^ Department of Digestive Surgery Estaing University Hospital Clermont Ferrand France; ^40^ Department of Radiology, ICO René Gauducheau Cancer Centre Saint Herblain France; ^41^ Department of Digestive Surgery Purpan University Hospital Toulouse France; ^42^ Department of Digestive Surgery Centre Hospitalier Lyon Sud, Pierre Bénite Lyon France; ^43^ Department of Medical Oncology Claudius Regaud Institute IUTC Toulouse France; ^44^ Department of Radiology, Hospital of Perpignan Perpignan France; ^45^ Department of Surgical Oncology, François Baclesse Comprehensive Cancer Centre Caen France; ^46^ Department of Surgical Oncology Bergonie Institute Bordeaux France; ^47^ Department of Digestive Surgery Minjoz University Hospital Besançon France; ^48^ Department of Radiology Robert Debré University Hospital Reims France; ^49^ Department of Radiology Montpied University Hospital Clermont Ferrand France; ^50^ Department of Pathology, Centre Hospitalier Lyon Sud, Pierre Bénite Lyon France; ^51^ Department of Digestive Surgery Lyon Sud University Hospital Lyon France; ^52^ Department of Pathology Lariboisière University Hospital Paris France; ^53^ Department of Digestive Surgery Robert Debré University Hospital Reims France; ^54^ Department of Radiology, ICO René Gauducheau Cancer Centre Saint Herblain France; ^55^ Department of Radiology, François Baclesse Comprehensive Cancer Centre Caen France; ^56^ Department of Surgical Oncology Paoli Calmettes Institute Marseilles France; ^57^ Department of Pathology Lorraine Institute of Oncology, Vandoeuvre les Nancy France; ^58^ Hospices Civils de Lyon, Pôle Information Médicale Evaluation Recherche, Unité de Recherche Clinique Lyon France; ^59^ Surgical Oncological and Digestive Unit Lariboisière University Hospital Paris France; ^60^ Department of Digestive Surgery Angers University Hospital Angers France; ^61^ Department of Radiology Curie Institute Paris France; ^62^ Department of Surgical Oncology Lorraine, Institute of Oncology Vandoeuvre les Nancy France; ^63^ Department of Surgical Oncology Curie Institute Paris France; ^64^ Hospices Civils de Lyon, Pôle Information Médicale Evaluation Recherche, Unité de Recherche Clinique Lyon France; ^65^ Department of Surgery, Léon Bérard Comprehensive Cancer Centre Lyon France; ^66^ Department of Pathology Claudius Regaud Institute IUTC Toulouse France; ^67^ Department of Surgery Louis Mourier University Hospital Colombes France; ^68^ Gynaecology and Obstetrics Ward, Mother and Child Pole, Poitiers University Hospital University of Poitiers Poitiers France; ^69^ Department of Surgical Oncology, Val d'Aurelle Montpellier Cancer Centre Montpellier France; ^70^ Department of Digestive Surgical Oncology University Hospital of Dijon Dijon France; ^71^ Department of Digestive Surgery Minjoz University Hospital Besançon France; ^72^ Imagerie Nucléaire de l'Ouest Lyonnais et de l'Ain (INOLA), Lyon France; ^73^ Department of Surgery, Léon Bérard Comprehensive Cancer Centre Lyon France; ^74^ Department of Digestive Surgery Estaing University Hospital Clermont Ferrand France; ^75^ Department of Digestive Surgery Timône University Hospital Marseilles France; ^76^ Surgical Oncological and Digestive Unit Lariboisière University Hospital Paris France; ^77^ Department of Pathology Paoli Calmettes Institute Marseilles France; ^78^ Department of Digestive Surgery St Etienne University Hospital St Etienne France; ^79^ Department of Digestive Surgical Oncology University Hospital of Dijon Dijon France; ^80^ Department of Pathology, François Baclesse Comprehensive Cancer Centre Caen France; ^81^ Department of Radiology Centre Hospitalier Lyon Sud, Pierre Bénite, Lyon France; ^82^ Department of Medical Oncology, ICO René Gauducheau Cancer Centre Saint Herblain France; ^83^ Department of Digestive Surgery Lyon Sud University Hospital Lyon France; ^84^ Department of Radiology Curie Institute Paris France; ^85^ Department of Surgical Oncology, Val d'Aurelle Montpellier Cancer Centre Montpellier France; ^86^ Department of Radiology Lyon Sud University Hospital Lyon France; ^87^ Department of Pathology, Hospital Saint Antoine Assistance Publique – Hôpitaux de Paris (AP HP), Paris France; ^88^ Department of Radiology, Val d'Aurelle Montpellier Cancer Centre Montpellier France; ^89^ Department of Surgical Oncology, ICO René Gauducheau Cancer Centre Saint Herblain France; ^90^ Department of Gastroenterology Nantes University Hospital Nantes France; ^91^ Department of Digestive Surgery Charles Nicolle University Hospital Rouen France; ^92^ Department of Pathology Minjoz University Hospital Besançon France; ^93^ Department of Digestive Surgery Lyon Sud University Hospital Lyon France; ^94^ Department of Radiology Poitiers University Hospital Poitiers France; ^95^ Department of Pathology, ICO Paul Papin Cancer Centre Angers France; ^96^ Hospices Civils de Lyon, Pôle Information Médicale Evaluation Recherche, Unité de Recherche Clinique Lyon France; ^97^ Department of Digestive and General Surgery, Georges Pompidou European Hospital Paris France

## Abstract

**Background:**

Pseudomyxoma peritonei (PMP) is a rare clinical condition characterized by mucinous ascites, typically related to appendiceal or ovarian tumours. Current standard treatment involves cytoreductive surgery (CRS) and hyperthermic intraperitoneal chemotherapy (HIPEC), but recurrences occur in 20–30 per cent of patients. The aim of this study was to define the timing and patterns of recurrence to provide a basis for modifying follow‐up of these patients.

**Methods:**

This observational study examined a prospectively developed multicentre national database (RENAPE working group) to identify patients with recurrence after optimal CRS and HIPEC for PMP. Postoperative complications, long‐term outcomes and potential prognostic factors were evaluated.

**Results:**

Of 1411 patients with proven PMP, 948 were identified who had undergone curative CRS and HIPEC. Among these patients, 229 first recurrences (24·2 per cent) were identified: 196 (20·7 per cent) occurred within the first 5 years (early recurrence) and 30 (3·2 per cent) occurred between 5 and 10 years. Three patients developed a first recurrence more than 10 years after the original treatment. The mean(s.d.) time to first recurrence was 2·36(2·21) years. Preoperative chemotherapy and high‐grade pathology were significant factors for early recurrence. Overall survival for the entire group was 77·9 and 63·1 per cent at 5 and 10 years respectively. The principal site of recurrence was the peritoneum.

**Conclusion:**

Recurrence of PMP was rare after 5 years and exceptional after 10 years.

## Introduction

Pseudomyxoma peritonei (PMP) is a condition characterized by mucinous ascites, which leads to abdominal distension, pain, bowel obstruction and anorexia. It results from extensive mucin secretion by a primary tumour in the peritoneal cavity, most commonly originating in the appendix or ovary^1^, but occasionally from mucinous colorectal tumours^1^, ^2^, ^3^. Treatment for PMP has evolved over the past two decades, and now ideally involves complete cytoreductive surgery combined with hyperthermic intraperitoneal chemotherapy (CRS plus HIPEC). When performed in a high‐volume centre, this combination can achieve overall survival (OS) rates of over 60 per cent at 10 years^4^, although, PMP being a heterogeneous disease, survival varies according to disease aggressiveness, primary pathology and the extent of tumour removal during the initial surgery^5^ ^6^. Surveillance is warranted as patients who develop a recurrence may be amenable to repeat surgery, leading to extended survival^7^ ^8^.

Patterns of recurrence in these slow‐growing tumours are currently not well defined, and optimal surveillance has not been standardized. The aim of the present study was to evaluate long‐term recurrence patterns in PMP treated with optimal CRS + HIPEC, to provide a better definition of the follow‐up required after apparent complete disease eradication.

## Methods

A prospective multicentre database (RENAPE working group) was used to identify all patients who had undergone surgery for PMP between 1993 and 2015^9^. The study was conducted in accordance with the tenets of the Declaration of Helsinki. The diagnosis of PMP was based on preoperative CT, operative findings and pathological confirmation. Preoperative data included patient demographics, origin of the primary tumour, number and type of previous interventions, and the use and type of systemic neoadjuvant therapy. Operative data included the Peritoneal Cancer Index (PCI), calculated during the operation^10^, size of residual lesions, duration of the surgery and the type of HIPEC used. Pathological analyses included histological grade (according to the WHO 2010 classification) and lymph node involvement^6^ ^11^. Tumours were graded as described by Carr^11^. Postoperative systemic treatments, long‐term survival and recurrence were recorded.

All patients analysed were treated with optimal CRS, and HIPEC was administered with either a closed or an open technique, as described previously^12^. Patients were excluded from analysis when they underwent a palliative debulking procedure. The quality of CRS was defined according to the Sugarbaker completeness of cytoreduction (CC) score^10^: CC‐0, no macroscopic residual tumour; CC‐1, residual tumour smaller than 2·5 mm; CC‐2, residual tumour between 2·5 and 25 mm; and CC‐3, residual tumour greater than 25 mm. When the score was CC‐0 or CC‐1, the CRS was considered complete. To achieve optimal CRS, patients underwent multiple peritonectomies and visceral resection of involved organs. For patients who had multiple CRS + HIPEC treatments, only the first treatment was included in the analysis. Patients were excluded when the follow‐up was insufficient for this analysis (less than 5 years).

Patients were followed up for recurrence according to the individual institutional guidelines. Follow‐up for patients with low‐grade tumours tended to include physical examination, estimation of cancer markers, and CT or MRI 6 months after surgery and every year up to 10 years. Follow‐up for patients with high‐grade tumours was generally followed in a manner similar to protocols used for colorectal adenocarcinoma with physical examination, estimation of cancer markers and CT every 3 months in the first 2 years, every 6 months for the next 3 years, and then annually for life. Recurrences were confirmed with CT, abdominal MRI or PET, according to previously published criteria^13^. When the diagnosis could not be ascertained with radiology, exploratory laparoscopy with biopsy was performed to confirm recurrence.

Three patient groups were created for comparative purposes: patients with no recurrence after follow‐up of at least 10 years, those with early recurrence within 5 years, and those with late recurrence between 5 and 10 years. Recurrences at more than 10 years of follow‐up were excluded from the statistical analysis.

### Statistical analysis

All statistical analyses were performed with SAS® 9.3 software (SAS Institute, Cary, North Carolina, USA). Descriptive data are expressed as mean(s.d.), results for quantitative variables as median (range or 95 per cent c.i.) values, and qualitative data as the numbers with percentages. χ^2^ or Fisher's exact test was performed to evaluate unpaired data. For paired data, ANOVA was performed. The Kruskal–Wallis test was used when there was non‐normality. *Post hoc* analyses were performed with the Tukey, Games–Howell or Dunn test to identify paired groups with statistically significant differences. Estimates of survival were calculated using the Kaplan–Meier method and compared with the log rank test. Significance was set at *P* < 0·050.

## Results

**Figure 1:**
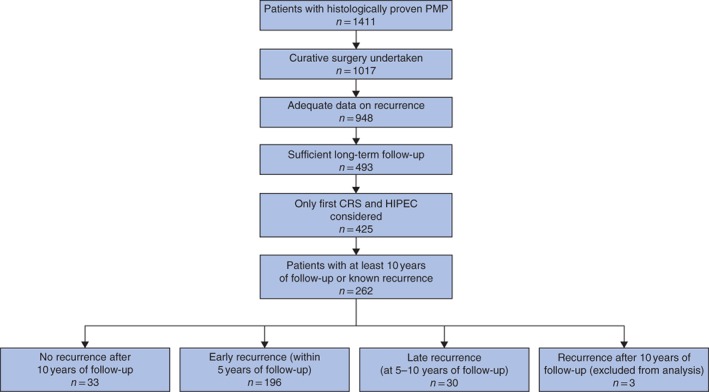
Flow chart of patient selection and study groups selected for analysis of pseudomyxoma peritonei (PMP). CRS, cytoreductive surgery; HIPEC, hyperthermic intraperitoneal chemotherapy

Of a total of 1411 PMP treatments during the study period, 394 patients who either underwent incomplete cytoreduction or did not receive HIPEC were excluded. A further 69 patients with missing data for recurrence were also excluded. A total of 948 patients met the inclusion criteria, of whom 229 (24·2 per cent) developed a recurrence (*Fig*. 1). Among the recurrences, 196 (85·6 per cent) occurred before the 5‐year follow‐up, 30 (13·1 per cent) between the 5‐year and 10‐year follow‐up, and three (1·3 per cent) after the 10‐year follow‐up (excluded from the statistical analysis). After optimal CRS + HIPEC, the mean time to first recurrence was 2·36(2·21) years (*Table* 1).

**Table 1:** Sites and time to recurrence in patients with recurrent pseudomyxoma peritonei

	All recurrences (*n* = 226)*	Early recurrence (*n* = 196)	Late recurrence (*n* = 30)*	*P* ‡
Type of recurrence				0·331
Peritoneal	148 of 225 (65·8)	129 (65·8)	19 of 29 (66)	
Extraperitoneal†	45 of 225 (20·0)	37 (18·9)	8 of 29 (28)	
Both	32 of 225 (14·2)	30 (15·3)	2 of 29 (7)	
Time to recurrence (years)				
Mean(s.d.)	2·36(2·21)	1·63(1·15)	7·15(1·30)	
Median (range)	1·53 (0·11–9·91)	1·24 (0·11–4·93)	6·96 (5·14–9·91)	

Values in parentheses are percentages unless indicated otherwise.

* Data on type of recurrence were missing for one patient.

† Extraperitoneal sites included liver, lung, lymph node, pleura and bone.

‡ χ^2^ test.

Of the three comparator groups created, there were 33 patients with no recurrence after follow‐up of at least 10 years, 196 who developed early recurrence within 5 years and 30 who developed late recurrence between 5 and 10 years (*Fig*. 1).

Patient characteristics are presented in *Table* 2. In the vast majority of patients (94·8 per cent), PMP was of appendiceal origin. The three patient groups underwent similar numbers of previous surgical treatments. Those with no recurrence after 10 years had a lower rate of preoperative systemic chemotherapy (18 per cent) than the other two groups (41·5 per cent for early and 28 per cent for late recurrence; *P* = 0·019). Postoperative systemic chemotherapy rates were similar between the groups. The WHO pathological classification of PMP indicated significantly more high‐grade disease in the early recurrence group (77·6 per cent) compared with late recurrence (48 per cent) and no recurrence (40 per cent) groups (*P* < 0·001). Intraoperative PCI values were significantly different between no recurrence and early recurrence groups (11 *versus* 24 respectively; *P* = 0·046).

**Table 2:** Clinicopathological and morbidity characteristics of patients with pseudomyxoma peritonei

	No. of patients* (*n* = 259)	No recurrence (*n* = 33)	Early recurrence (*n* = 196)	Late recurrence (*n* = 30)	*P* §
Age at surgery (years)†	53·9(11·6)	51·6(9·7)	54·9(11·8)	50·1(11·2)	0·052¶
Sex ratio (F : M)	153 : 106	22 : 11	116 : 80	15 : 15	0·405
Site of primary lesion					1·000
Appendix	221 of 233 (94·8)	29 of 30 (97)	163 of 173 (94·2)	29 (97)	
Ovary	9 of 233 (3·9)	1 of 30 (3)	7 of 173 (4·0)	1 (3)	
Other	3 of 233 (1·3)	0 of 30 (0)	3 of 173 (1·7)	0 (0)	
Previous surgery					
Laparoscopy	87 (33·6)	10 (30)	68 (34·7)	9 (30)	0·802
Laparotomy	66 (25·5)	7 (21)	48 (24·5)	11 (37)	0·302
Cytoreduction	90 (34·7)	13 (39)	69 (35·2)	8 (27)	0·550
Preoperative chemotherapy	95 of 257 (37·0)	6 (18)	81 of 195 (41·5)	8 of 29 (28)	0·020
PCI‡	23 (2–36)	11 (3–36)	24 (2–36)	16 (3–33)	0·046¶
Duration of surgery (min)‡	480 (90–875)	420 (150–690)	480 (95–875)	420 (90–765)	0·285¶
CC score					0·502
CC‐0	201 (77·6)	28 (85)	149 (76·0)	24 (80)	
CC‐1	58 (22·4)	5 (15)	47 (24·0)	6 (20)	
WHO classification					< 0·001
Low grade	62 of 199 (31·2)	15 of 25 (60)	33 of 147 (22·4)	14 of 27 (52)	
High grade	137 of 199 (68·8)	10 of 25 (40)	114 of 147 (77·6)	13 of 27 (48)	
Postoperative chemotherapy	36 of 258 (14·0)	2 (6)	32 (16·3)	2 of 29 (7)	0·178
Major complications	127 of 257 (49·4)	15 (45)	96 of 194 (49·5)	16 (53)	0·822

* With percentages in parentheses unless indicated otherwise values are

† mean(s.d.) and

‡ median (range). PCI, Peritoneal Cancer Index; CC, completeness of cytoreduction.

§ χ^2^ or Fisher's exact test, except

¶ ANOVA or Kruskal–Wallis test.

The mean time to recurrence was 1·63 years in the early recurrence group and 7·15 years in the late recurrence group, but the type of recurrence (peritoneal or extraperitoneal) was not significantly different between groups (*Table* 1). The peritoneum was the most frequent recurrence site (65·8 per cent for early and 66 per cent for late recurrence). Extraperitoneal sites were implicated in 18·9 per cent of early recurrences and 28 per cent of late recurrences. Both peritoneal and extraperitoneal sites were involved in 15·3 per cent of early recurrences and 7 per cent of late recurrences. Sites of recurrence were not significantly different between the groups when defined by the timing of the recurrence (*P* = 0·331) (*Table* 1). The organs involved in extraperitoneal recurrence included liver, lung, lymph node, pleura and bone, in descending order of frequency. There was no difference in recurrence site between tumour grades (*Table* 3).

**Table 3:** Site of recurrence according to pathological tumour grade

	All patients with recurrence (*n* = 173)*	Low grade (*n* = 46)	High grade (*n* = 127)	*P* †
Type of recurrence				0·620
Peritoneal	109 (63·0)	28 (61)	81 (63·8)	
Extraperitoneal	37 (21·4)	12 (26)	25 (19·7)	
Both	27 (15·6)	6 (13)	21 (16·5)	

Values in parentheses are percentages.

* Data on type of recurrence were missing for 53 patients.

† χ^2^ test.

**Figure 2:**
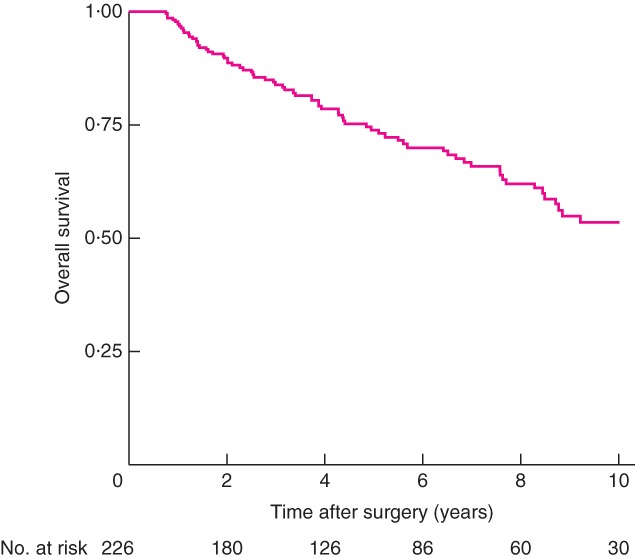
Kaplan–Meier curve for overall survival of patients with recurrent pseudomyxoma peritonei following cytoreductive surgery plus hyperthermic intraperitoneal chemotherapy

The entire group had 5‐ and 10‐year OS rates of 77·9 and 63·1 per cent respectively. After excluding patients with no recurrence, to determine survival among patients with recurrence, the 5‐ and 10‐year OS rates were 73·8 and 53·6 per cent respectively (*Fig*. 2).

**Figure 3:**
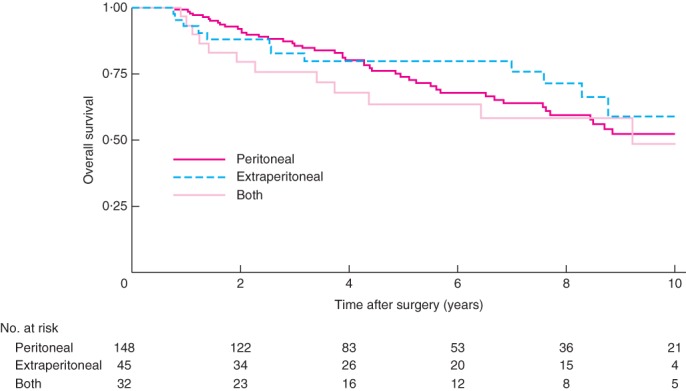
Kaplan–Meier curve for overall survival of patients with recurrent pseudomyxoma peritonei following cytoreductive surgery plus hyperthermic intraperitoneal chemotherapy, according to site of recurrence. *P* = 0·570 (log rank test)

When OS rates were analysed according to site of recurrence there was no significant difference in survival among patients with recurrence that occurred within the peritoneum, outside the peritoneum or at both sites (*Fig*. 3).

**Figure 4:**
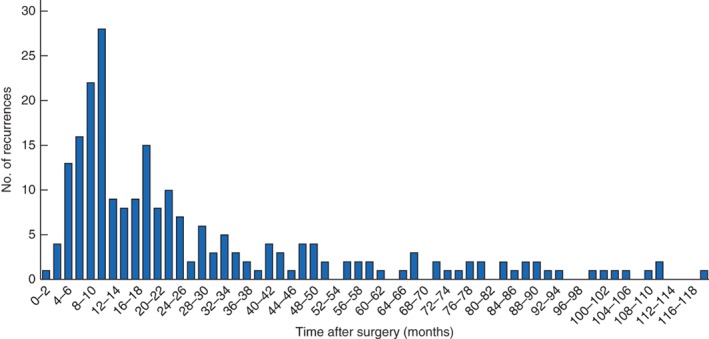
Recurrence of pseudomyxoma peritonei according to time of detection after surgery

The precise timing of recurrences, indicated by the number of recurrences in each 2‐month interval, is shown in *Fig*. 4.

## Discussion

The major finding of this study was the confirmation that about 25 per cent of patients treated with optimal CRS + HIPEC experienced a recurrence. PMP pathology and the use of preoperative chemotherapy were significant prognostic factors for recurrence. Although the mean time to recurrence was nearly 2·5 years, 14·4 per cent of patients (33 of 229) experienced a recurrence after 5 years. The OS rate of patients with a recurrent PMP who underwent a second optimal cytoreduction and HIPEC treatment was similar to that reported previously for patients treated for a first PMP^14^. The PCI score and site of recurrence did not significantly impact on the timing of recurrence.

The present study focused on patients who underwent optimal CRS + HIPEC. Of the 948 patients treated with this regimen, 229 (24·2 per cent) had a recurrence during follow‐up, similar to the 26·8 per cent recurrence rate reported by the Basingstoke group^8^ for 712 patients who received optimal CRS + HIPEC. Among recurrences in the present study, 85·6 per cent occurred within 5 years. In contrast, in a large population of patients with colorectal cancer, late recurrences (more than 5 years) following a curative resection accounted for only 4·3 per cent of all recurrences, and late recurrences were more likely to occur with less aggressive primary tumours^15^.

The largest international PMP registry^14^ has defined predictors of poor disease‐free survival as: previous systemic chemotherapy, high histopathological grade, major postoperative complications, high PCI, and no HIPEC after CRS. Those markers were consistent with the present results, where patients with aggressive histology (high grade) were more likely to experience early recurrence (before 5 years), and patients with low‐grade disease more likely to experience late recurrence.

For most types of peritoneal metastasis, a high intraoperative PCI has been associated with a poor prognosis^15^, ^16^, ^17^. This is not always the case, however, according to a Peritoneal Surface Oncology Group International review^14^, in which patients with mucinous disease of the peritoneum were considered to represent a distinct group that exhibited high survival rates, despite a very high PCI. The present results did not show a statistically significant association between the intraoperative PCI and timing of recurrence, although the intraoperative PCI score was greater in the early than in the late recurrence group (24 *versus* 16 respectively). This may reflect the small sizes of the comparator groups.

Previous systemic chemotherapy is another factor associated with poor prognosis. In the present study, the vast majority (82 per cent, 27 of 33) of patients with no recurrence after 10 years had not received preoperative chemotherapy. The proportion of patients who had received preoperative chemotherapy was significantly higher in the early recurrence than in the late recurrence group (41·5 *versus* 28 per cent). This might reflect a more aggressive pathology or a higher tumour burden that led the physician to determine a need for chemotherapy before surgery. Alternatively, the association between early recurrence and preoperative chemotherapy could be that chemoresistant tumour cell clones were selectively spared after systemic chemotherapy. This possibility suggests that the timing and the route of administration of chemotherapy may be important factors in preventing chemoresistant cell selection.

The location of recurrence was not influenced by the timing of recurrence. Recurrences were exclusively peritoneal in approximately two‐thirds of each group. Only about one‐quarter of patients in the early and late recurrence groups had an isolated extraperitoneal recurrence. A recent study^7^ reported that, in patients treated with curative intent for a first PMP recurrence after optimal CRS + HIPEC, the 5‐year OS rate was 83 per cent, compared with only 27 per cent in patients with extensive disease not amenable to optimal CRS + HIPEC. The present results were consistent with those from that study: among all patients who had a recurrence, 5‐ and 10‐year OS rates were 73·8 and 53·6 per cent respectively, compared with their reported rates of 68 and 61 per cent^7^. These findings emphasize the need for early detection of recurrence, as a good outcome is achievable when recurrent disease is resectable.

The ideal follow‐up regimen after CRS + HIPEC for PMP has not been standardized. Exposure to radiation, as a result of multiple CT scans, has been shown to be associated with an increased risk of secondary malignancy, particularly in younger patients^18^. Abdominal MRI can provide adequate detection of peritoneal mucinous accumulations, and the results seemed better than those achieved with CT in some centres^13^. Abdominal MRI may be an alternative to CT for follow‐up imaging, particularly in patients with low‐grade PMP, in whom the risk of extraperitoneal recurrence is low^14^ ^19^. Most recurrences in the present series occurred within the first 2 years after treatment, supporting the need for frequent surveillance during this interval.

This study has limitations. The inclusion period was long (23 years, from 1993 to 2015) and survival has improved over time as techniques have been modified^20^. In 1992, few centres performed CRS. Follow‐up methods have also changed, such as the inclusion of MRI. The greater sensitivity of MRI in detecting recurrence may have affected the apparent distribution of recurrences between the follow‐up time intervals defined in this study. HIPEC regimens and exposure times varied in different centres. Lack of standardization may have been associated with variations in patterns of recurrence between centres that could not be identified. As with all retrospective designs, sample size and missing data may have introduced bias.

The outcomes of CRS + HIPEC for PMP are improving and encouraging, particularly after optimal resection of the disease. As one in four patients experienced recurrence and more than 10 per cent of recurrences occurred after 5 years of follow‐up, it seems appropriate to follow these patients for at least 10 years. The ideal surveillance regimen remains to be defined, but the present results support combinations of CT and abdominal MRI for at least 10 years, conducted more frequently in the first 2 years, and focusing on abdominal recurrences that might be amenable to repeat surgery.

## Collaborators

Members of the RENAPE Network include: J. Abba (Department of Digestive Surgery, Grenoble University Hospital, Grenoble, France); K. Abboud (Department of General Surgery, Centre Hospitalier Universitaire, Jean Monnet University, Saint Étienne, France); M. Alyami (Department of Digestive Surgery, Lyon‐Sud University Hospital, Lyon, France); C. Arvieux (Department of Digestive Surgery, Grenoble University Hospital, Grenoble, France); G. Averous (Department of Pathology, Hautepierre University Hospital, Strasbourg, France); N. Bakrin (Department of Digestive Surgery, Lyon‐Sud University Hospital, Lyon, France); G. Balagué (Department of Radiology, Claudius Regaud Institute, Toulouse, France); V. Barrau (Department of Radiology, Centre Cardiologique du Nord, Saint Denis, France); H. Ben Rejeb (Department of Pathology, Bergonie Institute, Bordeaux, France); J.‐M. Bereder (Department of Digestive Surgery, Archet 2 University Hospital, Nice, France); I. Berton‐Rigaud (Department of Digestive Oncology, Institut de Cancérologie de l'Ouest (ICO) René Gauducheau Cancer Centre, Saint‐Herblain, France); F. Bibeau (Department of Pathology, Côte de Nacre University Hospital, Caen, France); I. Bonnefoy (Department of Digestive Surgery, Lyon‐Sud University Hospital, Lyon, France); D. Bouzard (Department of Surgery, Louis Mourier University Hospital, Colombes, France); I. Bricault (Department of Radiology, Grenoble University Hospital, Grenoble, France); C. Caramella (Department of Radiology, Gustave Roussy Institute, Villejuif, France); S. Carrère (Department of Surgical Oncology, Val d'Aurelle Montpellier Cancer Centre, Montpellier, France); C. de Chaisemartin (Department of Surgical Oncology, Paoli Calmettes Institute, Marseilles, France); M. Chassang (Department of Radiology, Archet 2 University Hospital, Nice, France); A. Chevallier (Department of Pathology, Archet 2 University Hospital, Nice, France); T. Courvoisier (Department of Digestive Surgery, Poitiers University Hospital, Poitiers, France); P. Dartigues (Department of Pathology, Gustave Roussy Institute, Villejuif, France); A. Dohan (Department of Radiology, Lariboisière University Hospital, Paris, France); C. Eveno (Surgical Oncological and Digestive Unit, Lariboisière University Hospital, Paris, France); M. Faruch‐Bilfeld (Department of Radiology, University Hospital of Toulouse, Toulouse, France); G. Ferron (Department of Surgical Oncology, IUCT Oncopole, Toulouse, France); J. Fontaine (Department of Pathology, Lyon‐Sud University Hospital, Lyon, France); L. Fournier (Department of Radiology, Georges Pompidou European Hospital, Paris, France); E. Gabiache (Department of Radiology, Claudius Regaud Institute – l'Institut Universitaire du Cancer de Toulouse (IUCT), Toulouse, France); J. Gagniere (Department of Digestive Surgery, Estaing University Hospital, Clermont‐Ferrand, France); D. Geffroy (Department of Radiology, ICO René Gauducheau Cancer Centre, Saint‐Herblain, France); L. Ghouti (Department of Digestive Surgery, Purpan University Hospital, Toulouse, France); F.‐N. Gilly (Department of Digestive Surgery, Centre Hospitalier Lyon‐Sud, Pierre‐Bénite, Lyon, France); L. Gladieff (Department of Medical Oncology, Claudius Regaud Institute IUTC, Toulouse, France); A. Guibal (Department of Radiology, Hospital of Perpignan, Perpignan, France); J.‐M. Guilloit (Department of Surgical Oncology, François Baclesse Comprehensive Cancer Centre, Caen, France); F. Guyon (Department of Surgical Oncology, Bergonie Institute, Bordeaux, France); B. Heyd (Department of Digestive Surgery, Minjoz University Hospital, Besançon, France); C. Hoeffel (Department of Radiology, Robert Debré University Hospital, Reims, France); C. Hordonneau (Department of Radiology, Montpied University Hospital, Clermont‐Ferrand, France); S. Isaac (Department of Pathology, Centre Hospitalier Lyon‐Sud, Pierre‐Bénite, Lyon, France); P. Jourdan‐Enfer (Department of Digestive Surgery, Lyon‐Sud University Hospital, Lyon, France); R. Kaci (Department of Pathology, Lariboisière University Hospital, Paris, France); R. Kianmanesh (Department of Digestive Surgery, Robert Debré University Hospital, Reims, France); C. Labbé‐Devilliers (Department of Radiology, ICO René Gauducheau Cancer Centre, Saint‐Herblain, France); J. Lacroix (Department of Radiology, François Baclesse Comprehensive Cancer Centre, Caen, France); B. Lelong (Department of Surgical Oncology, Paoli Calmettes Institute, Marseilles, France); A. Leroux‐Broussier (Department of Pathology, Lorraine Institute of Oncology, Vandoeuvre‐les‐Nancy, France); Y. Lherm (Hospices Civils de Lyon, Pôle Information Médicale Evaluation Recherche, Unité de Recherche Clinique, Lyon, France); R. Lo Dico (Surgical Oncological and Digestive Unit, Lariboisière University Hospital, Paris, France); G. Lorimier (Department of Digestive Surgery, Angers University Hospital, Angers, France); C. Malhaire (Department of Radiology, Curie Institute, Paris, France); F. Marchal (Department of Surgical Oncology, Lorraine Institute of Oncology, Vandoeuvre‐les‐Nancy, France); P. Mariani (Department of Surgical Oncology, Curie Institute, Paris, France); E. Mathiotte (Hospices Civils de Lyon, Pôle Information Médicale Evaluation Recherche, Unité de Recherche Clinique, Lyon, France); P. Meeus (Department of Surgery, Léon Bérard Comprehensive Cancer Centre, Lyon, France); E. Mery (Department of Pathology, Claudius Regaud Institute IUTC, Toulouse, France); S. Msika (Department of Surgery, Louis Mourier University Hospital, Colombes, France); C. Nadeau (Gynaecology and Obstetrics Ward, Mother and Child Pole, Poitiers University Hospital, University of Poitiers, Poitiers, France); S. Nougaret (Department of Surgical Oncology, Val d'Aurelle Montpellier Cancer Centre, Montpellier, France); P. Ortega‐Deballon (Department of Digestive Surgical Oncology, University Hospital of Dijon, Dijon, France); B. Paquette (Department of Digestive Surgery, Minjoz University Hospital, Besançon, France); O. Pellet (Imagerie Nucléaire de l'Ouest Lyonnais et de l'Ain (INOLA), Lyon, France); P. Peyrat (Department of Surgery, Léon Bérard Comprehensive Cancer Centre, Lyon, France); D. Pezet (Department of Digestive Surgery, Estaing University Hospital, Clermont‐Ferrand, France); N. Pirro (Department of Digestive Surgery, Timône University Hospital, Marseilles, France); M. Pocard (Surgical Oncological and Digestive Unit, Lariboisière University Hospital, Paris, France); F. Poizat (Department of Pathology, Paoli Calmettes Institute, Marseilles, France); J. Porcheron (Department of Digestive Surgery, St Etienne University Hospital, St Etienne, France); P. Rat (Department of Digestive Surgical Oncology, University Hospital of Dijon, Dijon, France); P. Rousselot (Department of Pathology, François Baclesse Comprehensive Cancer Centre, Caen, France); P. Rousset (Department of Radiology, Centre Hospitalier Lyon‐Sud, Pierre‐Bénite, Lyon, France); H. Senellart (Department of Medical Oncology, ICO René Gauducheau Cancer Centre, Saint‐Herblain, France); M. Serrano (Department of Digestive Surgery, Lyon‐Sud University Hospital, Lyon, France); V. Servois (Department of Radiology, Curie Institute, Paris, France); O. Sgarbura (Department of Surgical Oncology, Val d'Aurelle Montpellier Cancer Centre, Montpellier, France); A. Skanjeti (Department of Radiology, Lyon‐Sud University Hospital, Lyon, France); M. Svrcek (Department of Pathology, Hospital Saint‐Antoine Assistance Publique – Hôpitaux de Paris (AP‐HP), Paris, France); R. Tetreau (Department of Radiology, Val d'Aurelle Montpellier Cancer Centre, Montpellier, France); E. Thibaudeau (Department of Surgical Oncology, ICO René Gauducheau Cancer Centre, Saint‐Herblain, France); Y. Touchefeu (Department of Gastroenterology, Nantes University Hospital, Nantes, France); J.‐J. Tuech (Department of Digestive Surgery, Charles Nicolle University Hospital, Rouen, France); S. Valmary‐Degano (Department of Pathology, Minjoz University Hospital, Besançon, France); D. Vaudoyer (Department of Digestive Surgery, Lyon‐Sud University Hospital, Lyon, France); S. Velasco (Department of Radiology, Poitiers University Hospital, Poitiers, France); V. Verriele‐Beurrier (Department of Pathology, ICO Paul Papin Cancer Centre, Angers, France); L. Villeneuve (Hospices Civils de Lyon, Pôle Information Médicale Evaluation Recherche, Unité de Recherche Clinique, Lyon, France); F. Zinzindohoue (Department of Digestive and General Surgery, Georges Pompidou European Hospital, Paris, France).
